# Improving Quality of Life in Nursing Homes: The Structured Resident Interview Approach

**DOI:** 10.1155/2014/892679

**Published:** 2014-10-09

**Authors:** Howard B. Degenholtz, Abby L. Resnick, Natalie Bulger, Lichun Chia

**Affiliations:** University of Pittsburgh, 130 DeSoto Street, Pittsburgh, PA 15261, USA

## Abstract

The quality of life (QOL) of the approximately 1.5 million nursing facility (NF) residents in the US is undoubtedly lower than desired by residents, families, providers, and policy makers. Although there have been important advances in defining and measuring QOL for this population, there is a need for interventions that are tied to standardized measurement and quality improvement programs. This paper describes the development and testing of a structured, tailored assessment and care planning process for improving the QOL of nursing home residents. The Quality of Life Structured Resident Interview and Care Plan (QOL.SRI/CP) builds on a decade of research on measuring QOL and is designed to be easily implemented in any US nursing home. The approach was developed through extensive and iterative pilot testing and then tested in a randomized controlled trial in three nursing homes. Residents were randomly assigned to receive the assessment alone or both the assessment and an individualized QOL care plan task. The results show that residents assigned to the intervention group experienced improved QOL at 90- and 180-day follow-up, while QOL of residents in the control group was unchanged.

## 1. Background

This paper describes a novel assessment and care planning system for improving quality of life (QOL) in nursing homes. The Quality of Life Structured Resident Interview and Care Plan (QOL.SRI/CP) builds on a decade of research on measuring QOL and is designed to be easily implemented in any US nursing facility. The materials are compatible with the nursing home Minimum Data Set (MDS) 3.0. Instructions have been developed to allow for staged and iterative implementation.

QOL for people living in nursing facilities can be understood as a subjective assessment of the outcome of the clinical care, housing, and other services provided by the facility [1]. This concept of QOL is broader than that of health related quality of life (HRQOL), used in a lot of health care research [2, 3]. From the perspective of the individual resident, the concept of QOL refers to their perceptions of their own lives, as a function of their own personal preferences, personality, and life history as experienced in the daily environment of a nursing facility. Since nursing facilities structure not only the medical care but also the housing and social context, the definition of QOL for this population therefore incorporates the myriad ways that everyday life is facilitated and constrained by policies, procedures, and interactions with staff.

The QOL of the approximately 1.5 million nursing facility (NF) residents in the US is undoubtedly lower than desired by residents, families, providers, and policy makers [4]. Although the landmark Institute of Medicine report on nursing facility quality [5] and the subsequent 1987 legislative reforms placed high priority on QOL, much of the regulatory focus over the subsequent two decades was geared toward measuring and ensuring quality of care. One major outcome of those reforms was the development of the resident assessment instrument (also referred to as the MDS) which all NFs that accept Medicaid payment must use to collect data at regular intervals on all residents [6]. The RAI was designed primarily as a tool for* clinical* care planning and thus covers physical function, cognitive function, and other health care needs in detail [7]. The data derived from the RAI, known as the Minimum Data Set (MDS), are submitted to state licensure and certification agencies and subsequently to the Centers for Medicare and Medicaid Services (CMS) and serve as the main source of information for nursing home staff, policy makers, and researchers about the condition of nursing home residents. The data from the MDS have been used to develop indicators of quality of care for benchmarking and internal quality improvement and public reporting [8, 9]. The development of consumer friendly “report cards” is intended to use market forces to increase visibility and accountability for quality and further spur quality improvement efforts [10–12].

The recent revision of the MDS to version 3.0 has made major strides towards increasing attention paid to resident QOL [13]. Two changes are especially relevant. First, the designers have placed priority on resident self-report relative to observation and proxy report by incorporating “resident voice” in the assessment process. The general principle is that, for sections related to mood, pain, and preferences, the default expectation is to ask residents directly. Staff or family members can serve as a proxy only if the resident is unable to communicate or if an interpreter is not available [14, Appendix  D]. The second is the inclusion of two sets of questions about “daily preferences (F0400)” and “activity preferences (F0500)” [14]. The inclusion of these items is designed to produce information about residents' preferences that can be used as a guide to create individualized care plans. All nursing homes are required to have multidisciplinary care plans for each resident which serves as the primary documentation of all daily care and services. Under the regulations, care plans are developed based on information collected with the RAI and are reviewed and updated on regular basis (typically 90 days unless there is a change in status).

The critical role of the assessment process as part of individualized care planning cannot be understated. The CMS State Operations Manual [15] provides interpretive guidance for the survey and certification process and specifically highlights residents' right to choose activities and schedules consistent with their interests and make choices about their lives. The guidance also identifies the expectation that facilities identify each resident's interests and needs and involve the resident in activities that appeal to his or her interests. To be in compliance, staff needs to recognize and assess preferences and define and implement activities (including but not limited to formal and scheduled group activities) in accordance with resident needs and goals.

It is therefore imperative that NFs have the “technology” to determine resident preferences and incorporate those preferences into the resident's care plan. The wording of the items in MDS 3.0 Section F is intended to identify issues that residents find “important” for their daily lives. The MDS 3.0 manual notes that these items are not meant to be all-inclusive [14]. In addition to breadth, what is missing from the MDS 3.0 items is an approach to eliciting two important factors crucial to care planning. First, there needs to be a way to elicit whether residents' appraisal of their daily experience meets their expectations, given that they find a particular issue important. Second, in order to develop a truly individualized plan of care, staff needs to have a way to learn the content of resident's preferences. In order to meet the regulatory and ethical expectations of respecting residents' rights of self-determination, staff need to consistently learn what, when, how, and with whom individual residents want to live their daily lives. The MDS 3.0 does not provide the detailed prompts or questions to address this expectation.

## 2. Methods

We developed a structured resident interview and care planning system for nursing home residents and tested it in a longitudinal randomized, controlled trial at three nursing homes. The following sections describe the quality of life assessment, care plan, and all evaluation procedures.

Every aspect of the assessment and care planning materials was extensively and iteratively pilot tested. The procedure was to develop a draft assessment and have two interviewers (a former director of social service and a recent social work graduate) conduct a series of interviews with a small sample of nursing residents from one facility. After each sample, the study team reviewed the results and made revisions to the approach. In this way, we were able to streamline the assessment process and simplify the scoring. The final version is described below.

The study was conducted at three not-for-profit nursing homes in the Pittsburgh area. One facility was part of a regional faith-based chain. Two were owned by a not-for-profit medical system: one in an urban setting and one on a suburban campus that also included personal care homes and independent living apartments. All three facilities had skilled units dedicated to postacute care; however residents living on those units were excluded from the study. The number of beds averaged 161 (range from 137 to 182). Each had relatively stable leadership during the study period.

### 2.1. QOL Structured Resident Interview

The QOL.SRI consists of two main components: the Domain Questionnaire (DQ) and the in-depth follow-up. The DQ contains 69 closed-ended items drawn from previous research to define and measure quality of life among nursing home residents [16]. The items are organized into 12 domains: comfort, security, food enjoyment, privacy, meaningful activities, religious practices, relationships, functional competence, dignity, individuality, autonomy, and spiritual well-being. Based on extensive pilot testing, items related to religious practices were separated from meaningful activities to create a new domain area that can be easily skipped for residents who report that they do not practice a religion. Each item was rated using an unfolded four-point scale. Residents were first asked a yes or no question, followed by a frequency question: “yes” was followed up with “always or often” and “no” was followed up with “rarely or never.” This approach reduces the cognitive burden of using the full four-point scale [13] and improves participation rates [17].

Residents who provide a response that indicates “poor” quality of life (e.g., indicating they never find it easy to make friends) are asked to rate the importance of that issue. The importance rating follows the response categories used in the MDS 3.0 (very important, somewhat important, not very important, not important at all, and important but cannot do or no choice). This approach minimizes respondent burden by focusing on the information needed to make decisions about care planning around issues where there is a need for improvement.

A simple algorithm was developed to calculate a priority score by multiplying the QOL rating for each item by the importance rating for that item. If no importance rating was obtained, the priority score was blank. The numbers were arranged so that lower QOL received a lower score and higher importance received a lower score. Thus the product of the two numbers could be interpreted as a “rank” wherein priority is given to items in declining order (i.e., 1st, 2nd, 3rd, etc.). This information was then used as a guide for in-depth probing.

Each item on the DQ has a corresponding set of in-depth (ID) follow-up questions (see Table 2 for a sample item). These are open-ended probes designed to elicit the “what, where, when, and with whom” for each topic. To reduce respondent burden, the assessor selected only the top 5 ranking items for in-depth follow-up. Based on our pilot testing, we observed that occasionally residents might mention issues that are not covered in the DQ items or make other comments that indicate that an issue is very important to them. In these cases, the assessor was allowed to select one “wild-card” item to follow-up on an issue that might not have scored in the top 5 but nonetheless seemed especially important to the resident.

### 2.2. QOL Care Plan

The QOL care planning component can be considered a standardized “tailored” intervention. All residents received a standardized assessment; however the actual questions and the intervention they received were tailored to their own individual preferences based on their responses to the assessment questions. The approach is similar to an adaptive questionnaire.

The assessor used the residents' responses to the open ended component of the QOL.SRI to develop a draft QOL care plan task. These tasks were written on a simple form that identified the issue of concern, the goal in terms of resident QOL, the staff involved, and the frequency with which the task should be accomplished. The assessor used the responses to the ID questions to select one specific issue to address. Care was taken to develop tasks that were practical and feasible for staff to execute without requiring substantial amounts of additional time or resources. Impossible requests (e.g., visit with a deceased relative) were not addressed; however, roommate changes were considered. The draft care plan task was brought to the unit manager and the appropriate department head (i.e., if a task involved activities or dietary staff, the corresponding person was involved) for their review and approval. Afterwards, the task was placed into the appropriate order book for the department.

All three facilities used electronic point-of-care systems for nurse aides. In two of the facilities, a reminder was placed into a voice-activated system that informed the aide to check a project-specific order book placed at the nurses' station. In the third facility a detailed description of the task was placed onto the touch screen kiosk. Thus, in all three facilities, the QOL care plan task was placed at the same level of accountability as other orders.

### 2.3. Recruitment, Randomization, and Interview Process

We obtained a census of all residents living in each of the three participating facilities in order to screen for study eligibility. We excluded residents who were receiving hospice services and rehabilitation, were not elderly, did not speak English, were under infection quarantine, or lived in a dementia special care unit. Letters were sent by the facilities to all residents and, if available, community residing spouses or people with power of attorney informing them of the study and providing instructions on how to opt out. Research staff approached residents using a verbal informed consent script that included multiple check points for understanding. Residents were asked to repeat back key elements of the informed consent disclosure, and if their responses were not correct then the assessor terminated the interview. We anticipated heterogeneity in the types of care plans that residents in the treatment condition would receive; therefore residents were randomized to treatment and control conditions using a 60/40 allocation.

Residents were assessed at baseline. The complete assessment was repeated at 90 and 180 days. Residents who were randomized to the treatment group had a QOL care plan task provided to nursing home staff for implementation after the baseline interview. After the 180-day follow-up interview, QOL care plan tasks were written for all residents (treatment and control) and provided to staff.

### 2.4. Analysis

To assess the comparability of the treatment and control groups, we constructed physical function and cognitive function scores based on data drawn from the nursing home Minimum Data Set. Physical function was computed as the sum of level of difficulty with 10 activities of daily living (moving from lying position, transfer, walking in room, walking in corridor, moving between room and corridor, moving on and off the unit, dressing, eating, using the toilet, and grooming) (ranged from 0 to 40). Cognitive function was computed as the sum of items measuring short-term and long-term memory and ability to make daily decisions (ranged from 0 to 5).

We calculated QOL scores for each of the 12 QOL domains measured in the assessment. The change from baseline to 90 days was calculated and averaged across treatment and control groups. To display the 180-day findings, we calculated the average “difference in difference” between treatment and control groups. This yields a positive value if the QOL in the treatment group is higher than the QOL in the control group.

## 3. Findings

### 3.1. Sample

**Figure 1:**
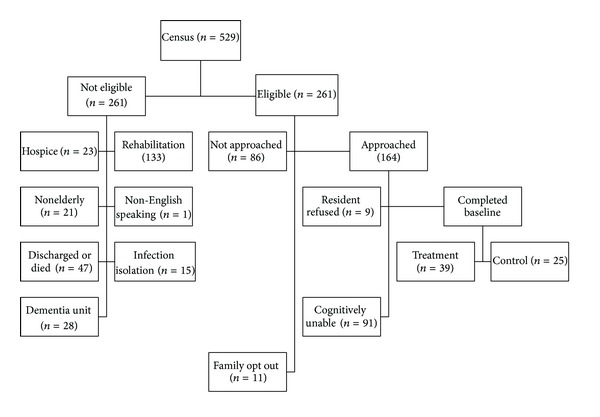
Sample recruitment.

The results of subject recruitment are shown in Figure 1. Only 11 family members opted out on behalf of eligible residents. A total of 164 residents were approached (86 additional residents were eligible but were not approached because we had reached our recruitment goal). A total of 64 residents were randomized using a 60/40 quota, yielding 39 in the treatment group and 25 in the control group. At the 90-day point, 12 residents from the treatment group were unavailable (3 were discharged, 4 had died, 2 were cognitively unable, 1 refused, 1 was quarantined, and 1 was physically unable to participate) and 6 from the control group were unavailable (2 were discharged, 2 had died, and 2 were cognitively unable). At the 180-day point, 5 residents from the treatment group were unavailable (1 was discharged, 3 were cognitively unable, and 1 was quarantined) and 4 from the control group were unavailable (1 had died, 1 was cognitively unable, and 2 refused). Our analysis is focused on those residents who were assessed at baseline and the 90-day assessment (a subset of whom were also assessed at 180 days).

At baseline, treatment and control groups were similar in terms of age (82.6 versus 82.4; *P* = .923), gender (*n* = 6; 15% male versus *n* = 5; 22% female; *P* = .363), and race (*n* = 7; 18% African American; *n* = 3; 13% African American; *P* = .466). Physical function scores were 22 and 24.0 in treatment and control (*P* = .266). Cognitive function scores were 1.5 and 1.6 in treatment and control (*P* = .843).

### 3.2. QOL Care Plans

Care plans were categorized based on the domain area of the issue that was being addressed.  Table 1 lists the types of care plans and provides examples. The most common care plans addressed functional competence, followed by meaningful activities and comfort. Although none of the care plans were based on a topic elicited during the autonomy section of the assessment, all are written with an “autonomy” focus. All of the care plans direct staff to ask residents if, how, or when they would like things done. There is also some overlap. For example, the QOL care plan related to religious practices has an element of functional competence. The QOL care plan related to security focuses on relationships with staff because the resident indicated that staff is not responsive to her requests related to personal care that is physically painful. The decision was made to focus on improving her relationship with staff in order to reduce the tension and distrust.

### 3.3. Change in 90-Day QOL Scores

**Figure 2:**
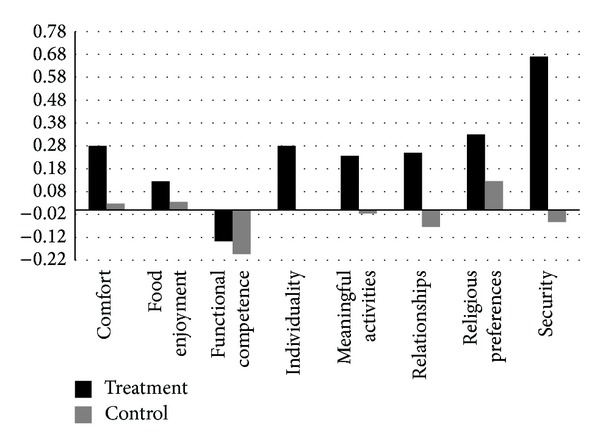
Change in quality of life domain scores by QOL care plan target.

We calculated the change in QOL domain score for each resident and grouped QOL care plan target area. For example, the 5 residents whose care plans were focused on a comfort issue are grouped together. For comparison, we calculated the change in each of the 8 targeted QOL domains in the control group. Thus, in the comparison group, only those residents whose QOL care plan targeted a particular domain are included in the change score, whereas all of the control group members are used for all of the comparison domain scores. This is presented graphically in Figure 2. The average change in QOL across all domains in the treatment group was .25 (SD .40), compared to a slight decline of −.02 (SD .39) in the control group. This comparison was statistically significant using a one-sided* t*-test (*P* = .014).

### 3.4. Change in 180-Day QOL Scores

**Figure 3:**
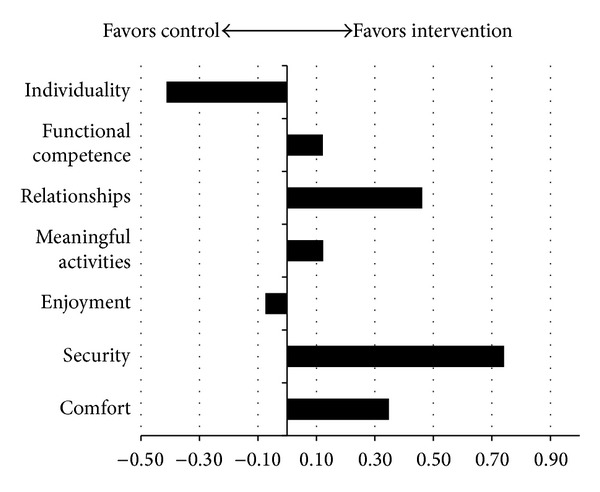
Difference between treatment and control at 180 days.

To examine the 180-day outcomes, we compared the change in the treatment group to the change in the control group on each domain score. This was calculated as the difference in difference; the resultant score is positive if the treatment group outperforms the control group and negative if the opposite is true.  Figure 3 shows the difference in differences. The average of the differences in difference scores at 180 days was .20 (SD = .41). This is statistically significantly different compared to zero using a one-sided* t*-test (*P* = .0346).

## 4. Discussion

This study demonstrated that an individualized assessment and care planning system, the QOL.SRI, can produce meaningful and lasting improvements in resident QOL. The system was developed to be straightforward to implement and provide a high level of accountability within a traditional nursing home management structure.

The intervention group experienced improvements in all QOL domains at 90 days with the exception of functional competence. This area is closely linked with physical function and can be expected to decline over time in this population. We note that the decrease in QOL scores in this domain was lower in the treatment group than in the control group (the small sample size precludes statistical significance testing of this comparison). At 180 days, the treatment group still experienced higher levels of QOL than the control group. The overall difference was slightly smaller and in two domains (individuality and food enjoyment) was actually negative. This is particularly important considering that the QOL care plan tasks were not updated or changed between 90 and 180 days. Thus, residents in the treatment group were still receiving the benefit of personalized attention to an issue raised approximately six months earlier.

Previous individual-level intervention research in the nursing home setting has focused on modifying specific factors related to the broad concept of QOL, such as autonomy [18, 19], psychological well-being [19], pain [20], physical function [21], or physical comfort (positioning) [22]. Certainly, reducing the use of physical restraints is clearly in service of improving QOL and has been shown to reduce injuries [23, 24]. Other studies have found that personal relationships [25] and quality of communication [26] between residents and staff can be enhanced and that simple interventions can improve the mealtime experience [27]. Activities programs such as gardening [28] demonstrate improvement in socialization and physical function. Life reminiscence [29] and programs, such as TimeSlips [30] which use creative storytelling with demented residents, have been shown to improve general QOL and reduce depressive symptoms [31, 32].

There have been several noteworthy facility-wide interventions that place priority on resident-centered care and have the potential to improve QOL. Wellspring [33] is a quality improvement program based on staff empowerment and resident directed. Anecdotal evidence suggests that residents in Wellspring facilities experienced improved QOL; however the evaluation did not include a quantitative measure of QOL. Likewise, the Green House model is a comprehensive alternative to the traditional nursing home, involving completely different architecture, staffing, and operations [34]. Results of the evaluation show that residents in the Green House experience better quality of life than those in traditional nursing homes (an average of .39 points on a 4-point scale) [35].

This study builds on previous research on measuring QOL that found that 91% of the variation in resident self-reported QOL scores can be attributed to differences among individual residents rather than between facilities [36]. Even though it is possible to rank order nursing homes based on resident QOL scores, the variation in ranks cannot be fully explained by organizational factors [37]. Taken together, these findings suggest that effective interventions need to be tailored to the individual resident. Facility-wide improvements are important, however, and should not be forgone. However, the best improvements are ones that enable staff to identify and act on individual residents' preferences.

The QOL.SRI takes about 30 minutes to administer. This is not a trivial commitment for most facilities, given the constant challenges of staffing and competing demands for limited resources. An important benefit of the system, in addition to improving individual resident QOL, is that it generates data about resident QOL that can be aggregated to the unit or facility level in order to track performance and support quality improvement efforts. These data can be compared to the experience of other facilities in order to benchmark improvement over time.

In this study, we repeated the assessment at 90 and 180 days in order to determine whether QOL improvements would persist. We had found that QOL changes reverted; we would be in a position to argue that these issues should be discussed more frequently with residents. However, the results were somewhat mixed on this point. Although residents continued to experience better QOL on average, anecdotally, several of the more cognitively intact residents indicated at the 180-day interview that they were expecting their care plans to be refreshed and asked why further improvements had not been made.

Facilities may elect to use the QOL.SRI on an annual basis. This would reduce the amount of data available to monitor individual and aggregate change. However, staff would be able to return to the answers to the ID component throughout the year and raise new issues at the quarterly care conference. This would likely provide a richer and more systematic source of information about resident preferences than is currently available and, as residents experience decline in their cognitive function, can serve as a historical record of their likes and dislikes.

### 4.1. Limitations

Resident QOL has been found to be associated with physical function, cognitive function, and various facility factors. Longitudinal research found that decline in resident health is associated with decline in QOL; however, the amount of variation explained by these factors is relatively small. The potential exists that residents in the treatment group experienced greater declines in such factors than the control group. Although the sample sizes were small, there is no evidence of differential decline over time.

The relatively small sample size limits our ability to conduct extensive multivariate or subgroup analyses. The study was constrained by the time required to recruit, randomize, and follow up on residents. The decision to exclude short-stay residents was based on the expectation that many would not be available for the 90-day follow-up. However, that decision eliminated a large number of potential residents from participation and limits the generalizability to the long-stay population. We were limited to three nonprofit nursing homes in the Pittsburgh area. A larger study with more facilities would be able to identify organizational factors associated with fidelity of implementation and outcomes.

The assessments were conducted and care plans were written by a member of the study team (NB). This individual had a similar profile to a nursing home social worker in terms of training and experience. After a short period, facility staff accepted her presence and treated her as a colleague. By the same token, residents came to identify her as the “QOL person.” Since her role and function were independent from the facility, it is possible that residents felt more comfortable discussing problems with care staff than they might have been with a nursing home employee.

## 5. Conclusion

The QOL.SRI provides a system for nursing homes to conduct a tailored and individualized assessment of resident QOL that can be used to inform care planning. The approach has been shown to lead to improvements in resident QOL. The materials are available without charge from a publicly accessible website: http://www.improvingqol.pitt.edu.

## Figures and Tables

**Table 1:** Description of sample.

Care plan domain	*N*	Examples
Functional competence	7	(i) Ask resident if she would like her bathroom straightened up (ii) When in resident's room at same time as resident, ask her if she would like anything moved within her reach

Meaningful activities	6	(i) Ask resident about current reading materials and if she would like new books or other reading materials (ii) When there is an activity involving cards (blackjack, etc.) invite resident to join

Comfort		(i) Ask resident if she would like her pillows or bed height adjusted
	5	(ii) When assisting resident with getting dressed, ask resident if she would like to have any extra layers on or nearby
		(iii) Each night ask resident if the temperature of her room is acceptable

Food enjoyment	4	Ask resident if her food is warm enough and offer to microwave it if cold

Relationships	2	During one-on-one visits with resident ask if she would like materials for her in-room activities

Religious preferences	1	Twice a week, ask resident if she needs any of her religious materials moved within her reach

Individuality	1	(i) Once a week, visit with resident to talk about prior life experiences such as military service (ii) When giving care to resident take extra five minutes to engage resident in a conversation about talking points in his room

Security	1	Three days a week, stop in to see resident in her room and engage her in a short conversation

**Table 2:** Sample QOL.SRI item.

Domain	Item	Importance	How often?	How important?	In-depth probes
Relationships	In the past month, have people who work here stopped just to have a friendly conversation with you?	[1.5] Yes	[4] Always [3] Often		
		[3.8] No	[2] Rarely [1] Never	[1] Very [2] Somewhat	
				[3] Not very [4] Not important [1.5] Important but cannot do (no choice)	Where do these conversations usually occur? When do these conversations occur? Who do you wish you could talk to more often?
